# Interstitial lung disease related to occupational hard metal exposure: two case reports

**DOI:** 10.1186/s13256-023-04043-4

**Published:** 2023-07-20

**Authors:** I-Fan Lin, Hsiao-Chin Shen, Shiou-Fu Lin, Ho-Chuen Chang, Tzu-Tao Chen

**Affiliations:** 1 Department of Occupational Medicine and Clinical Toxicology, Taipei Veterans General Hospital, Taipei, Taiwan; 2 Department of Medicine, Taipei Medical University, Taipei, Taiwan; 3 Department of Occupational Medicine, Shuang Ho Hospital, New Taipei City, Taiwan; 4 Department of Medical Education, Taipei Veterans General Hospital, Taipei, Taiwan; 5 Department of Chest Medicine, Taipei Veterans General Hospital, Taipei, Taiwan; 6 Department of Pathology, Shuang Ho Hospital, New Taipei City, Taiwan; 7 Department of Medical Imaging, Shuang Ho Hospital, New Taipei City, Taiwan; 8 Division of Pulmonary Medicine, Department of Internal Medicine, Shuang Ho Hospital, New Taipei City, Taiwan; 9 Graduate Institute of Clinical Medicine, College of Medicine, Taipei Medical University, Taipei, Taiwan; 10 Division of Thoracic Medicine, Department of Internal Medicine, School of Medicine, College of Medicine, Taipei Medical University, Taipei, Taiwan; 11 Division of Critical Care Medicine, Department of Emergency and Critical Care Medicine, Shuang Ho Hospital, New Taipei City, Taiwan

**Keywords:** Interstitial lung disease, Hard metal lung disease, Tungsten carbide, Case report

## Abstract

**Background:**

Hard metal lung disease (HMLD) is a relatively less known occupational interstitial lung disease, and instances of HMLD resulting from para-occupational exposure are rarely reported.

**Case presentation:**

This paper presents two cases of interstitial lung disease caused by exposure to hard metal. The first case involves a 37-year-old Taiwanese man who had worked at a grinder station for hard metal materials for 12 years without respiratory protective equipment. He experienced a dry cough and exertional dyspnea, and his chest imaging and pathology findings were consistent with the features of usual interstitial pneumonia. Analysis of his lung tissue revealed the presence of tungsten and cobalt. The second case involves a 68-year-old Taiwanese woman, the mother of the first patient, who had hand-washed her son’s workwear. She experienced a dry cough and had similar imaging findings to her son. After her son left his job, they both exhibited improved symptoms and lung functions with nintedanib treatment. These findings suggest a diagnosis of HMLD and interstitial lung disease resulting from para-occupational exposure to hard metal dust.

**Conclusions:**

The diagnosis of HMLD relies on obtaining a detailed occupational exposure history. If HMLD is diagnosed, discontinuing exposing to hard metal dusts can lead to improved lung function.

**Supplementary Information:**

The online version contains supplementary material available at 10.1186/s13256-023-04043-4.

## Background

Well-known occupation-related interstitial lung diseases (ILD) include silicosis, asbestosis, and coal workers’ pneumoconiosis [1]. Hard metal lung disease (HMLD) is a relatively less known but important cause of occupational ILD. Hard metals are composed of sintered tungsten carbide (80–90%) and cobalt (6–9%) and have been widely used in industry due to their extreme hardness and corrosion resistance [2, 3]. Exposure to hard metal dust occurs during the manufacture of hard metals and during the grinding of hard metal products [4]. The reports of HMLD dates back to 1940 [5]. Early studies reviewed the chest radiographs of hundreds of hard metal workers and estimated the prevalence of lung fibrotic change in hard metal workers at 2% to 18% [5].

In this paper, we present two cases of ILD diagnosed with high-resolution computed tomography (HRCT). The first case was exposed to hard metal dust while working. Video-assisted thoracoscopic surgery (VATS) was conducted and revealed tungsten and usual interstitial pneumonia (UIP) with fibrosis, leading to a diagnosis of HMLD. The second case was the first patient’s mother. After ruling out other possible causes, she was likely to have HMLD resulting from para-occupational exposure to hard metal dust in her son’s workwear. We discuss the effects of hard metal exposure cessation and nintedanib treatment on both patients.

## Case presentation

The cases are described according to the CARE guidelines [6].

A 37-year-old non-smoking Taiwanese man visited the hospital in December 2019 after an abnormal chest radiograph during a health examination earlier that year. He had suffered from a dry cough and dyspnea on exertion for more than six months. Physical examination revealed bilateral coarse crackles. No clubbing of fingers or cyanosis of lips and fingertips was observed.

This patient had worked at a grinder station for hard metal materials in a precision machinery factory without proper ventilation system or any respiratory protective equipment for 64 hours per week since 2007. His colleague who had worked with him for 5 years suffered from bronchitis and a cough.

**Fig. 1:**
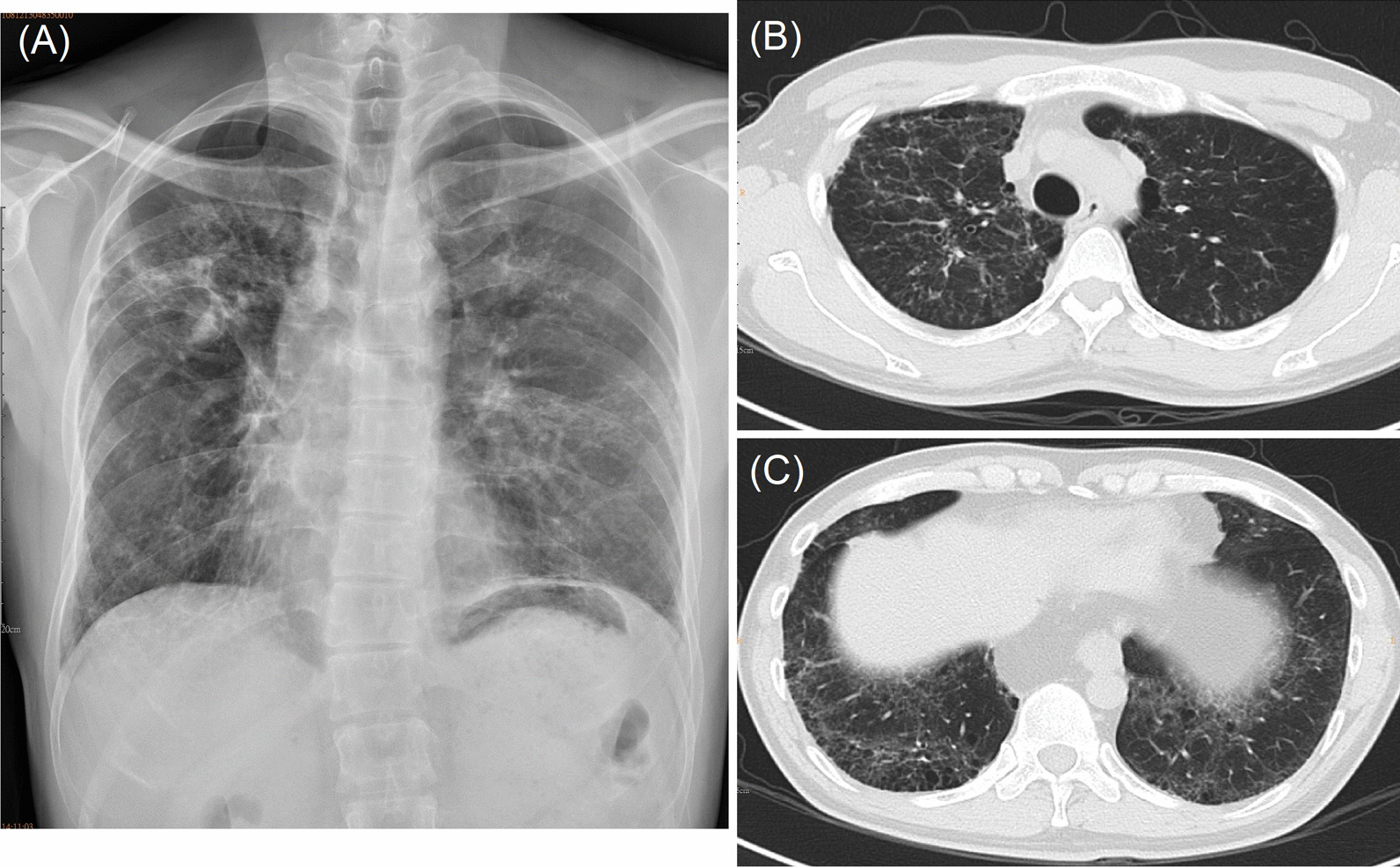
**A** The chest radiograph and **B** high-resolution computed tomography images (upper and lower lungs) of the first patient revealed nodular infiltration in the bilateral upper lungs, and **C** diffused honeycombing in the bilateral lung bases

A chest radiograph showed nodular infiltration with partial fibrosis in the right upper lobe, multiple tiny nodules in both the upper and lower left lung fields and honeycombing in bilateral lung bases (Fig. 1A). HRCT revealed a tree-in-bud pattern in bilateral upper lungs with bronchiectasis in both upper and lower lungs and diffused honeycombing in bilateral lung bases (Fig. 1B, C). Pulmonary function test demonstrated a tendency to restrictive ventilatory impairment with a forced vital capacity (FVC) at 74% of the predicted value, the forced expiratory volume in 1 s/forced vital capacity ratio (FEV1/FVC) at 0.91, the diffusing capacity of the lung for carbon monoxide (DLCO) at 46% of the predicted value. The walking distance in 6-min walking test was 491. His baseline oxygen saturation was 95%, and his oxygen saturation dropped to 93% during the 6-min walking test. Heart echogram showed normal pulmonary artery systolic pressure (Right Ventricular Systolic Pressure = 18 mmHg).

His sputum acid-fast stain and tuberculosis (TB) cultures came back negative. A screening for autoimmune diseases revealed positive anti-nuclear antibody (ANA) with mixed speckled pattern (1:40). The quantities of complement 3, complement 4, rheumatic factor, and anti-cyclic citrullinated peptide (anti-CCP) in his blood were all within the normal range.

Bronchoalveolar lavage results were negative in both Gram stain and acid fast stain, no growth of any micro-organism; the red blood cell count was 63/μl, white blood cell count was 740/μl, comprised of eosinophil 10% (ref: ≤1%), lymphocyte 18% (ref: 10–15%), neutrophil 6% (ref: ≤3%), which was considered as eosinophilic cellular pattern[7]. The cytologic evaluation of bronchial washing revealed only bronchial and inflammatory cells.

This patient had a spontaneous pneumothorax and underwent a video-assisted thoracic surgery (VATS) wedge resection of the right upper and lower lobes in March 2020. He was re-admitted to hospital in August 2020 due to recurrent right spontaneous pneumothorax and underwent VATS wedge resection of the right middle lung and pleurodesis.

**Fig. 2:**
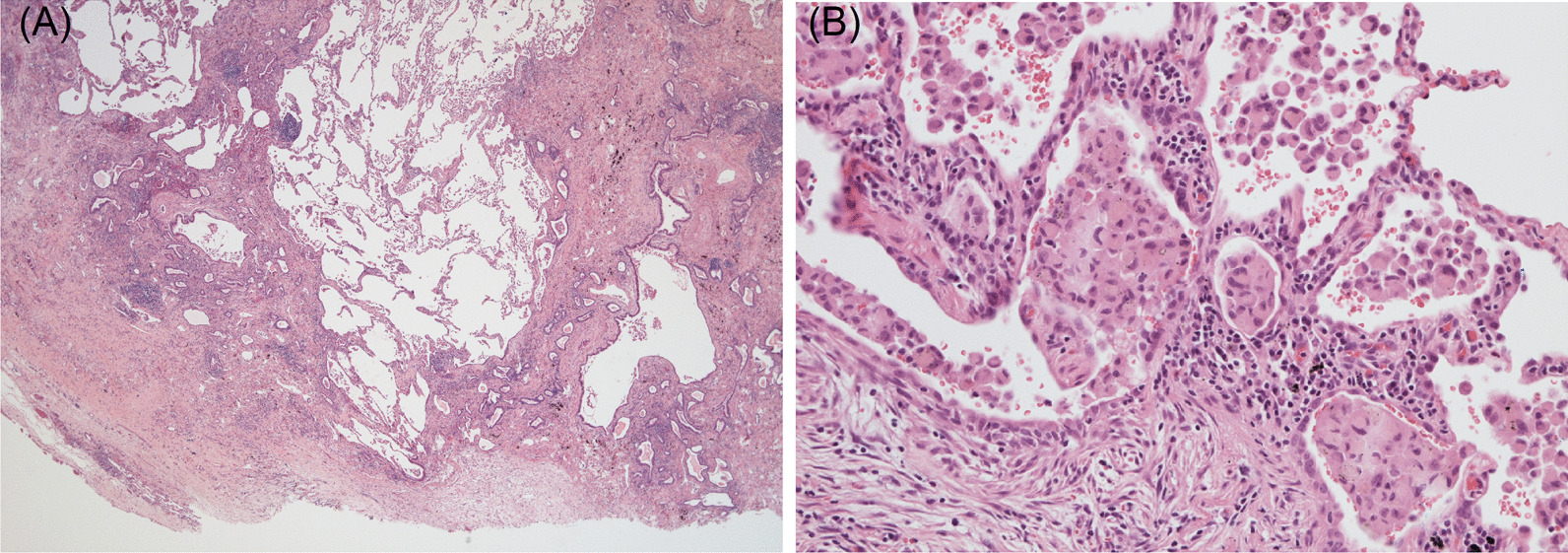
Sections of the lung tissue of the first patient. **A** showed architectural distortion with focal honeycombing in a subpleural distribution. **B** showed foci of aggregates of intra-alveolar pigmented macrophages and few giant cells

Sections of his right upper and lower lobes from VATS wedge resections revealed patchy fibrosis composed of dense collagen, fibroblast foci, and architectural distortion with focal honeycombing in a subpleural distribution (Fig. 2A). These findings suggest UIP. Tiny foci of aggregates of intra-alveolar pigmented macrophages and a few giant cells were found and suggestive of hard metal exposure (Fig. 2B). Inductively coupled plasma mass spectrometry (Agilent 7800 ICP-MS, Santa Clara, USA) analysis of his lung tissue revealed 0.231 ppb tungsten and 0.012 ppb cobalt.

**Fig. 3:**
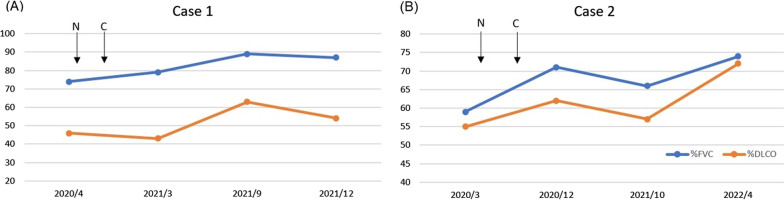
**A**, **B** showed the measured % forced vital capacity and diffusing capacity of the lung for carbon monoxide after nintedanib treatment for the first and second patients, respectively. ‘C’ stands for ‘cessation of exposure’, and ‘N’ stands for ‘nintedanib treatment’

This patient was diagnosed with HMLD with a UIP pattern and received nintedanib treatment (150 mg twice a day) since April 2020. He also stopped working at the factory after August 2020. His symptoms subsided, with an improvement in FVC and DLCO in the subsequent pulmonary function tests (Fig. 3A).

**Fig. 4:**
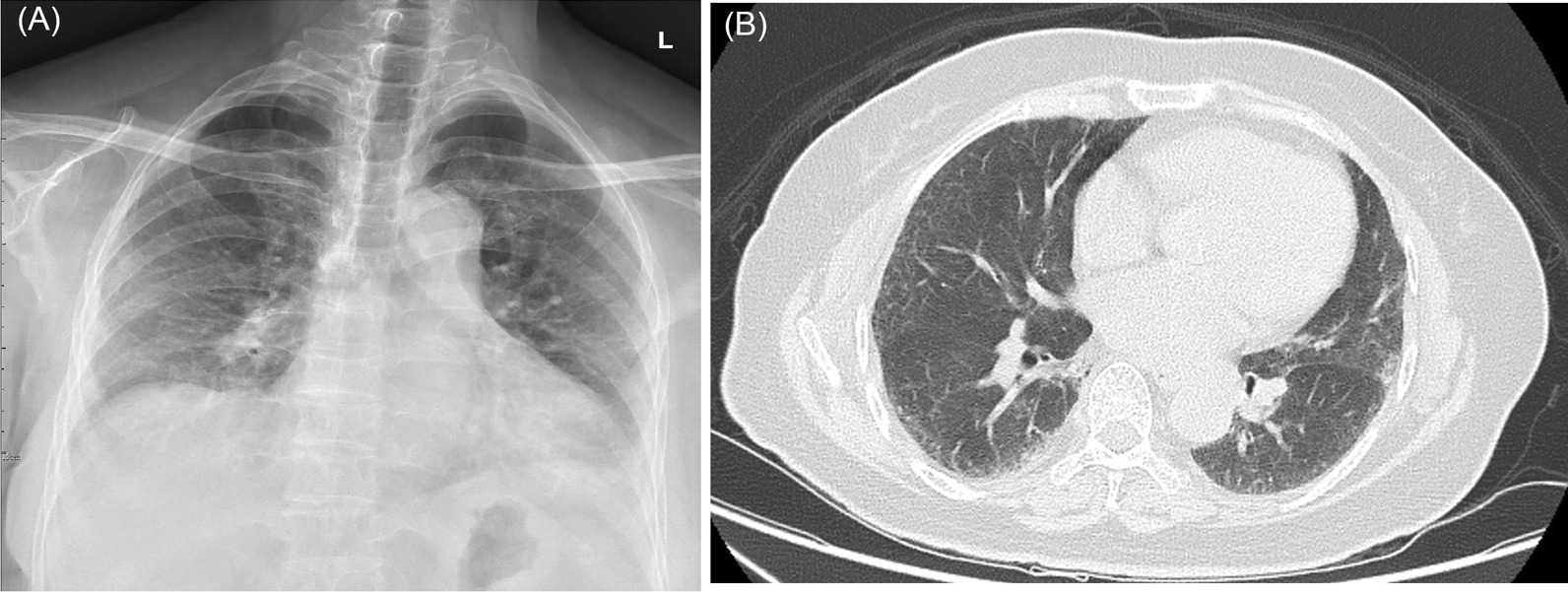
**A** The chest radiograph and **B** high-resolution computed tomography images (middle lungs) of the second patient revealed increased reticulonodular infiltrations in both lungs with honeycombing

His mother, aged 68, accompanied him to the hospital in Dec 2019 and complained of a dry cough and intermittent exertional dyspnea for 2 years. Chest radiograph showed increased reticulonodular infiltrations in both lungs (Fig. 4A). Physical examination revealed fine crackles in bilateral lower lobes. No clubbing of fingers or cyanotic of lips or fingertips was observed. Her oxygen saturation measured by pulse oximeter was 96%. HRCT showed diffused subpleural reticulations and ground-glass opacities with an apicobasal gradient and minimal basal lung honeycombing, consistent with a UIP pattern (Fig. 4B). Pulmonary function test showed a tendency to restrictive ventilatory impairment: FVC at 59% of the predicted value, FEV1/FVC at 0.99, and the DLCO at 55% of the predicted value. An examination of her auto-immune profile returned results that were all within the normal range. She never smoked, and she quit her job after getting married. She had hand-washed his son’s working wear every day. She also received nintedanib treatment (150 mg twice a day) since April 2020 (Fig. 3B). She reported that her symptoms improved after the treatment, but she discontinued the treatment in May 2020 due to side effects including serious diarrhea. Her symptoms recurred, and as a result she resumed nintedanib treatment in December 2020. She did not undergo bronchoalveolar lavage or a lung biopsy.

The first patient’s father and one of his brothers live with him and his mother in a high-rise apartment in a residential area. His father and his two brothers, including the one living with him, had no cough or any other related symptoms. He and his mother had no known food or drug allergies, and they did not have a history of other respiratory diseases.

## Discussion

These two cases demonstrate the importance of thorough history taking when it comes to differentiating the causes of ILD, including occupational exposure, family clusters, medication usage, tobacco exposure, mold exposure, and family history. ILD is a group of diseases comprising chronic inflammation within the lung and varying degrees of lung fibrosis [8]. Pathological and radiological examinations are important for ILD diagnosis, and medical history taking and serologic examinations are important for clarifying the cause of ILD.

In the two cases presented, there was no history of medication, smoking, or mold exposure prior to the diagnosis of ILD. In the first patient, the initial impression was idiopathic pulmonary fibrosis with UIP pattern in HRCT [9]. This patient had worked at a hard metal grinder station for 12 years, while the second patient had been exposed to the first patient’s clothes contaminated with hard metal dust for the same duration. These cases represent a family cluster and highlight the important role of para-occupational exposure in HMLD.

Serologic examinations showed no evidence of autoimmune-related ILD in the second case, while the first patient’s ANA test was positive but not strong enough (1:40) to diagnose autoimmune-related ILD. In addition, bronchoscopic examination of the first patient showed both eosinophilic and lymphocytic cellular pattern. Although the elevated percentage of lymphocytes indicated lymphocytic alveolitis (the percentage of lymphocytes exceeds 20%) [10–12], pathology examination did not support eosinophilic pneumonia.

In a previous cross-sectional study of 19 HMLD patients, most patients with a history of exposure to hard metal dust of less than 10 years (*N* = 15) had pathological findings showing giant cell interstitial pneumonia, and four patients who had hard metal dust for more than 10 years (*N* = 4) had pathological findings showing UIP [13]. The HRCT radiological findings of HMLD patients in past studies include reticular opacities, traction bronchiectasis, emphysema, and bulla [13, 14]. Our observations in these two cases are consistent with these previous reports.

Previous studies have shown that pulmonary function improved after cessation of hard metal dust exposure [15, 16]. However, pulmonary fibrosis progressed in 45% of the HMLD patients after the cessation of exposure has been reported [17]. Nintedanib was authorized for the management of idiopathic pulmonary fibrosis (IPF) in 2014. As pulmonary fibrosis progresses, a platelet-derived growth factor (PDGF) plays an important role in the proliferation and differentiation of lung fibroblasts. Nintedanib is a potent inhibitor of PDGF receptor (PDGFR) and has demonstrated its potential to reduce the decline of FVC and mortality [18, 19]. Nintedanib has also been employed in cases of interstitial lung disease (ILD) caused by systemic sclerosis in these years [20], and it has been suggested that nintedanib may be effective in treating other types of ILD based on recent studies [21, 22]. Therefore, although nintedanib is not a standard treatment option for occupational interstitial lung disease without lung fibrosis, it has been used to avoid progressive pulmonary fibrosis for occupational lung disease [23]. In this study, these two patients showed an overall trend of improvement in FVC and DLCO after the first case stopped hard metal dust exposure, although their imaging studies did not show significant improvement (see Additional file 1).

The strength of this report is that tungsten and cobalt were found in the first patient’s VATS specimens. The limitations of this report include the lack of hard metal dust exposure assessment in the first patient’s workplace or on his workwear and the absence of biological markers of hard metal dust in the second patient. The mechanisms that cause HMLD are not clear, although cobalt in the hard metals is believed to plays an important role in HMLD due to its allergic, teratogenic and carcinogenic effects [24]. Nevertheless, while in previous studies tungsten was detected in the lung tissue of most patients with HMLD, cobalt was detected in only some of them [13, 25]. The absence of cobalt from the lung tissue might be due to its solubility, but this observation also indicates the potential contribution of tungsten [17]. In addition, the synergic effect of cobalt and tungsten has also been suggested [26–29].

## Conclusions

The two cases presented here demonstrate the development of HMLD due to occupational and para-occupational exposure. Although both patients showed improvement after the cessation of hard metal dust exposure and with nintedanib treatment, appropriate measures to avoid occupational and para-occupational exposure such as workplace ventilation, the usage of respiratory protective equipment, and a change of clothes after work are necessary to prevent the development of these diseases.

## Supplementary Information

**Additional file 1:** The results of pulmonary function test and imaging series for Case 1 and Case 2, and the results of 6-min walking test and heart echo for Case 1.

## Data Availability

Most important data is presented in this paper. All clinical data before the consent forms were signed are available upon request.

## Abbreviations

ANA: Anti-nuclear antibody
FVC: Forced vital capacity
HMLD: Hard metal lung disease
HRCT: High-resolution computed tomography
ILD: Interstitial lung diseases
UIP: Usual interstitial pneumonia
PDGF: Platelet-derived growth factor
PFT: Pulmonary function test
TB: Tuberculosis
VATS: Video-assisted thoracoscopic surgery
