# Supplementary material for: Interstitial lung disease related to occupational hard metal exposure: two case reports
Source: J Med Case Rep. 2023 Jul 20;17:312. doi: 10.1186/s13256-023-04043-4 (PMC10357746; doi:10.1186/s13256-023-04043-4)

Additional file 1

Case 1: Pulmonary function test (2020/4/6)

|  | Measured value | % predicted value |
| --- | --- | --- |
| FVC | 3.01 | 74 |
| FEV1 | 2.73 | 80 |
| FEV1/FVC | 91% |  |
| PEF | 6.04 | 73 |
| PEF 25-75% | 3.27 | 88 |
| PEF 25% | 5.94 | 78 |
| PEF 50% | 4.88 | 110 |
| PEF 75% | 1.76 | 98 |
| TLC | 5.22 | 90 |
| VC | 3.01 | 74 |
| RV | 2.21 | 136 |
| DLCO | 11.0 | 46 |
| DLCO/VA | 2.86 | 63 |

Case 2: Pulmonary function test (2020/3/23)

|  | Measured value | % predicted value |
| --- | --- | --- |
| FVC | 1.36 | 59 |
| FEV1 | 1.35 | 73 |
| FEV1/FVC | 99% |  |
| PEF | 5.09 | 104 |
| PEF 25-75% | 2.95 | 151 |
| PEF 25% | 2.73 | 59 |
| PEF 50% | 5.07 | 186 |
| PEF 75% | 1.52 | 225 |
| TLC | 3.84 | 96 |
| VC | 2.30 | 72 |
| RV | 1.53 | 133 |
| DLCO | 9.0 | 55 |
| DLCO/VA | 4.06 | 110 |

Case 1 six minute walking test (2022/5/27)

O2 Sat%: 96-94

HR: 83-138

60%MHR-80%MHR: 109-145

Borg scale: 0-2

CAT(0-0-1-1-0-0-2-3): 7

Distance: 491

O2 consumption (estimated): 12.45ml/kg/min, 3.56 METs

FVC: 3.36 (L) 84 (% pred) Post exercise % change: -3.0 (%)

FEV1: 2.91 (L) 87 (% pred) Post exercise % change: -1.4 (%)

FEV1/FVC: 87%

Case 1 Heart Echo (2020/12/24)

AO root: 25 (20-37 mm)

LA: 25 (19-40 mm)

IVS: 10 (7-12 mm) 2D-Simpson: 69% (>50%)

LVEDD: 32 (35-52 mm)

LVPW: 10 (8-12 mm)

LVESD: 19 (20-35 mm)

RVSP/PASP: 18 mmHg

Case 1 Imaging series

2020/3/9 2020/7/6


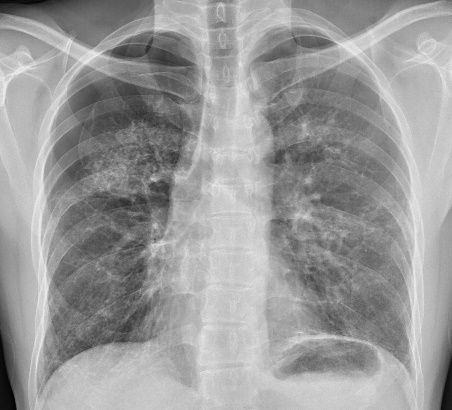

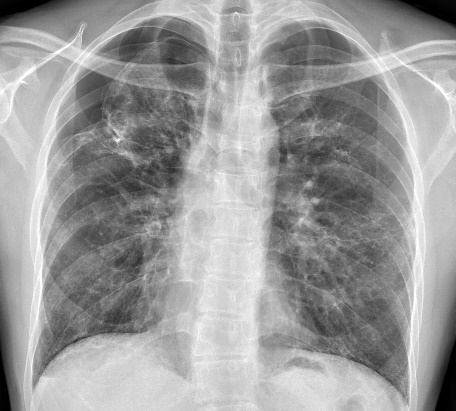


2020/8/3 2021/3/6 2021/8/26


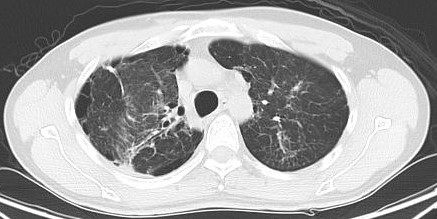

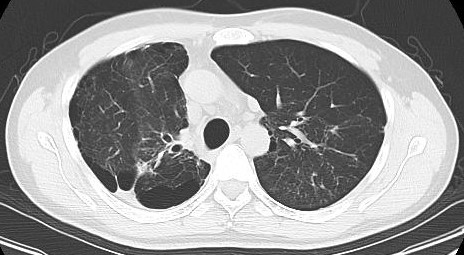

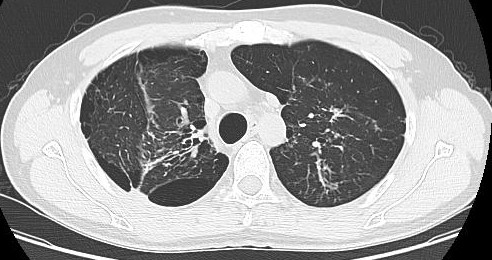


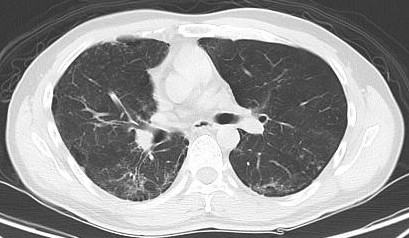

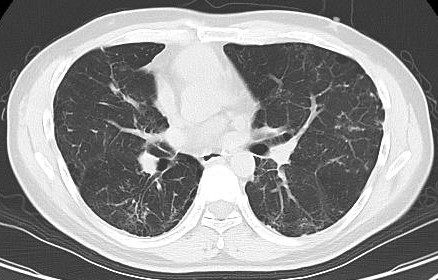

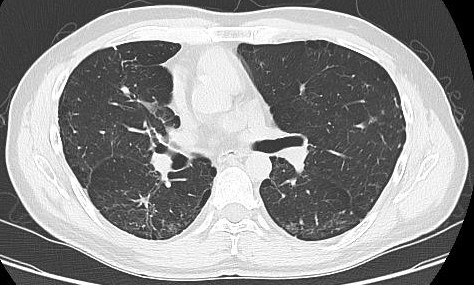


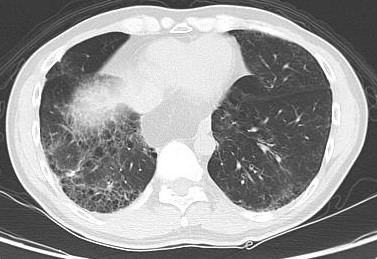

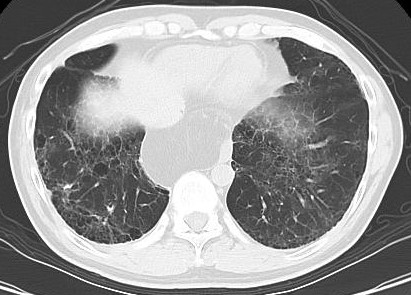

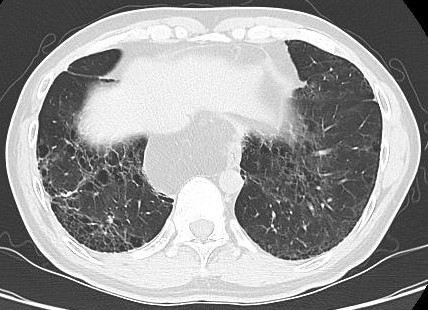


Case 2 Imaging series

2020/3/9 2020/12/14 2022/4/6


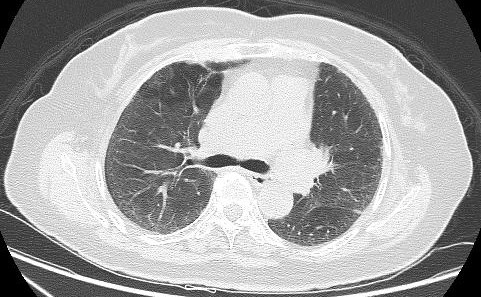

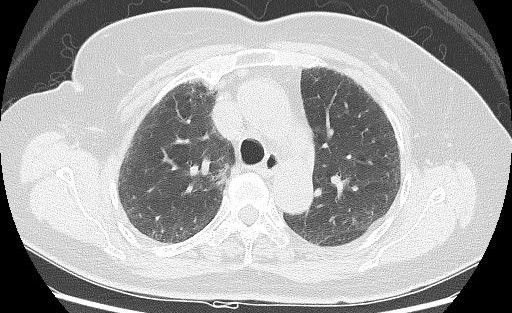

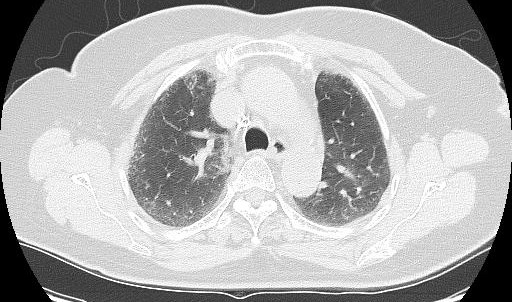


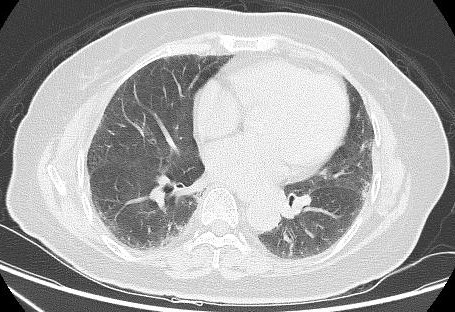

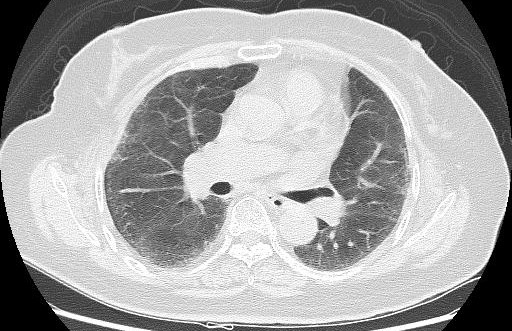

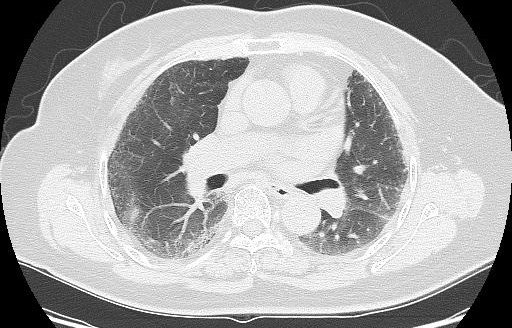


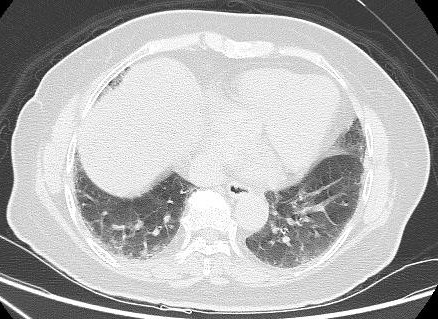

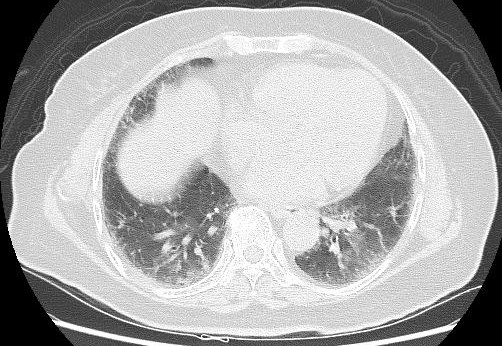

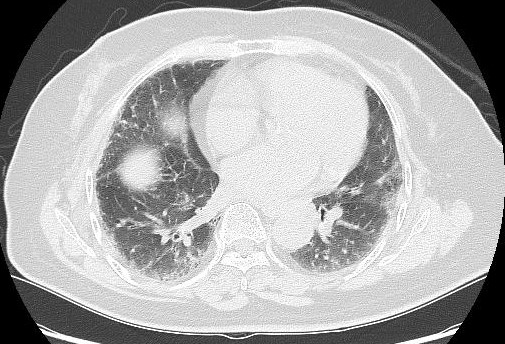

Supplement: Supplementary file 1 — Additional file 1: The results of pulmonary function test and imaging series for Case 1 and Case 2, and the results of 6-min walking test and heart echo for Case 1. [file 13256_2023_4043_MOESM1_ESM.docx]
